# Editorial: Emerging talents in genomic assay technology

**DOI:** 10.3389/fgene.2023.1259011

**Published:** 2023-09-11

**Authors:** Honey V. Reddi, Youri I. Pavlov

**Affiliations:** ^1^ Division of Precision Medicine and Cytogenetics, Department of Pathology, Medical College of Wisconsin, Milwaukee, WI, United States; ^2^ Eppley Institute for Research in Cancer and Allied Diseases, University of Nebraska Medical Center, Omaha, NE, United States

**Keywords:** biomarker discovery, next-generation sequencing (NGS), genomic assays, disease evaluation methods, single cell genomics, precision medicine

Precision medicine is an emerging science for the treatment and prevention of disease considering the variability in an individual’s genetic makeup in the context of environment and lifestyle. Precision medicine allows for the identification of genetic causation in the case of inherited disorders, the determination of variants for cancer diagnosis, and the treatment of disease as well as risk assessment for disease, enabling individualized or tailored applications for disease management. This is made possible by the advancements in genomic technologies that have significantly progressed in the last 2 decades, from genomics to multi-omics including proteomics and metabolomics.

The isolation of Taq polymerase in 1976 (Saiki et al., 1988) and the invention of PCR in 1985 (Mullis and Faloona, 1987) set the stage for the advent of molecular profiling in basic research. The development of Sanger sequencing in 1977 (Heather and Chain, 2016) stimulated the development of capillary sequencers in the sequencing of the human genome in 2003 (Heather and Chain, 2016). The last couple of decades have seen the field of genomic technologies grow by leaps and bounds with the advent of next-generation sequencing including long-read sequencing and single-cell sequencing (Shieh, 2023). This Research Topic showcases advancements in genomic technologies involving students, trainees, and post-doctoral fellows from the perspective of emerging talents in the field.

Single-cell sequencing (SCS) is a technique for sequencing the cellular genome, transcriptome, epigenome, proteomics, or metabolomics after the dissociation of tissues into single cells. In a translational research setting, Xu et al.demonstrate the use of single-cell RNA sequencing to demonstrate smooth muscle heterogeneity in aortic dissection, using a mouse deficient in apolipoprotein. In the context of tumors, SCS can dissect human tumors at a single-cell resolution, finely delineating different cell types and revealing the heterogeneity of tumor cells. Qin et al. summarize the basic process and development of single-cell sequencing technology and its increasing role in the field of hepatocellular carcinoma in a review. While the application of genomic technologies for discovery in translational research is the norm, the use of such applications for diagnostic, prognostic, or therapeutic applications in genomic medicine is the standard of care. Zhang et al., in their article, demonstrate the high specificity, efficiency, and safety (non-invasiveness) of using cell-free DNA for non-invasive prenatal testing (NIPT) to effectively improve the detection rate of common chromosomal aneuploidy, thereby reducing the occurrence of birth defects. The authors evaluate data from ∼69,000 maternal blood samples that were subjected to whole genome sequencing of ccfDNA.

Innovation in technology to improve specificity, sensitivity, and efficiency is key to the successful translation of any assay to the clinic. The detection of aneuploidies is not limited to pre-natal diagnosis, with a significant clinical utility being demonstrated in preimplantation genetic testing (PGT-A). Additionally, evaluating embryos for causative mutations for monogenic diseases (PGT-M) transferred from parents to children during the *in vitro* fertilization (IVF) treatment process is also well established. Yang et al. present a novel NGS-based methodology TAGs-seq PGT-A/M which allows for the simultaneous detection of monogenic diseases and genomic imbalances in one experiment using low-pass (∼30X) whole genome sequencing (WGS) and high-depth target enrichment sequencing (TES) reads, respectively (Yang et al., 2021). Lei et al. describe a novel DNA enrichment method—region-specific amplification (RSA) to identify break points in the 22q11.2 deletion by specific amplification of an approximately 1-Mb region where the breakpoint might exist. 22q11.2 deletion syndrome is a disorder caused when a small part of chromosome 22 is missing. Diagnosis is currently established by the identification of a heterozygous deletion at chromosome 22q11.2 through chromosomal microarray analysis or other genomic analyses. Using long-read sequencing with the Oxford Nanopore MinION sequencer, this article demonstrates that RSA can be used to not only sequence the target region 22q11.2 but could also be used for other hard-to-sequence parts of the genome. Similarly, Pei et al. show the application of a new PCR-based genome-walking method, fusion primer-driven racket PCR (FPR-PCR), for the reliable retrieval of unknown flanking DNA sequences. Genome-walking generally includes the generation of a genome library which is a cumbersome and labor-intensive process followed by PCR-based strategies which have low success rates, high background, and involve complex procedures. The FPR-PCR is demonstrated to be a high-efficiency genome-walking approach and a promising alternative to the available genome-walking protocols.

Last but not least, Bohm and Wyrick present a review of unique methods, such as CHIP-Seq, adduct-Seq, and Circle-Seq, for mapping both canonical and atypical UV-induced photoproducts across the genome. UV-induced DNA damage results in the formation of cyclobutane pyrimidine dimers (CPDs) which lead to C>T mutations and to mutations that give rise to both melanoma and non-melanoma skin cancers. However, recent studies have shown that UV-induced C>T mutations are not responsible for many of the key driver mutations that cause melanomagenesis but rather mutation classes with little to no prior link to UV-induced DNA damage, including T>C, T>A, and AC>TT, are the culprits, driving the need to further investigate the intricate relationship between UV exposure, DNA damage, and mutagenesis and forming the basis for this mini-review.

In conclusion, this Research Topic on ‘Emerging Talents in Genomic Assay Technologies’ showcases contributions by students, trainees, and post-doctoral fellows on different aspects of genomic technologies for basic, translational, and clinical research including their importance in the identification of genetic causation for inherited diseases and cancer. We believe that it is critical to give opportunity to the next-generation of scientists to showcase their findings, knowing that the need for precision medicine will continue to drive advancements in genomics technologies. We thank the contributing authors and hope that their articles are appreciated by their readers.
